# Never Fear, the Robot Is Here: Robotic Resection for a Giant Thymoma

**DOI:** 10.1016/j.atssr.2025.06.020

**Published:** 2025-07-22

**Authors:** Alison S. Baskin, Andrew D. Wisneski, Kirk D. Jones, Johannes R. Kratz, David M. Jablons

**Affiliations:** 1 Department of Surgery, University of California, San Francisco, San Francisco, California; 2 Department of Pathology, University of California, San Francisco, San Francisco, California

## Abstract

Thymomas are rare anterior mediastinal tumors that can grow large, compressing important thoracic structures. Complete surgical excision remains the “gold standard.” Whereas median sternotomy has traditionally been used, minimally invasive techniques are becoming increasingly favored. Recent studies highlight the safety and efficacy of robotic thymectomy; however, “large” tumors described in the literature have averaged 6 to 8 cm. We present the case of a 68-year-old woman with a 13-cm thymoma resected entirely robotically. Use of various port configurations and enhanced maneuverability of robotic platform instruments enabled adequate visualization and safe dissection. This case highlights that tumor size alone should not preclude robotic thymectomy.

Thymoma is a tumor arising from thymic epithelial cells in the anterior mediastinum, with an incidence of 0.13 per 100,000 persons per year.^1^ Despite its rarity, it is the most common anterior mediastinal mass. Thymomas are often asymptomatic and discovered incidentally; however, larger tumors or those with local invasion may cause compressive symptoms including chest pain, cough, and dyspnea. Paraneoplastic syndromes, such as myasthenia gravis, are also manifested in 30% to 50% of cases.^2^

Despite their generally indolent nature and variable biologic behavior, all thymomas are considered malignant.^3^ Thus, complete surgical excision remains the “gold standard.” Traditional approaches include median sternotomy, but minimally invasive options such as video-assisted thoracoscopic surgery (VATS) and robot-assisted VATS are becoming increasingly popular.^4^^,^^5^ Minimally invasive thoracic surgery is associated with faster recovery, reduced postoperative pain, and comparable oncologic outcomes.^6^^,^^7^

Although studies have highlighted the safety and efficacy of robotic thymectomy for select large tumors, most “large” tumors reported in the literature have averaged around 6 to 8 cm in size.^6^, ^7^, ^8^ In accordance with the CARE checklist, we report the successful robotic resection of a giant right-sided thymoma, highlighting key considerations for intraoperative management of a challenging surgical scenario.

A 68-year-old woman with history of right-sided breast cancer (treated with breast-conserving surgery and radiotherapy), asthma, and hyperlipidemia was incidentally found to have a right-sided chest mass during a coronary calcium scan. Computed tomography of the chest demonstrated a heterogeneously enhancing soft tissue mass measuring 12 × 9 × 10 cm, abutting the superior vena cava (SVC; Figure 1). A computed tomography–guided core needle biopsy had been performed at the referring hospital, confirming the diagnosis of thymoma. A minimally invasive approach would be attempted, with the patient counseled on the risk of conversion to open if visualization became challenging. In the weeks before surgery, progressive dyspnea and cough developed, believed to be symptoms related to airway compression.

**Figure 1:**
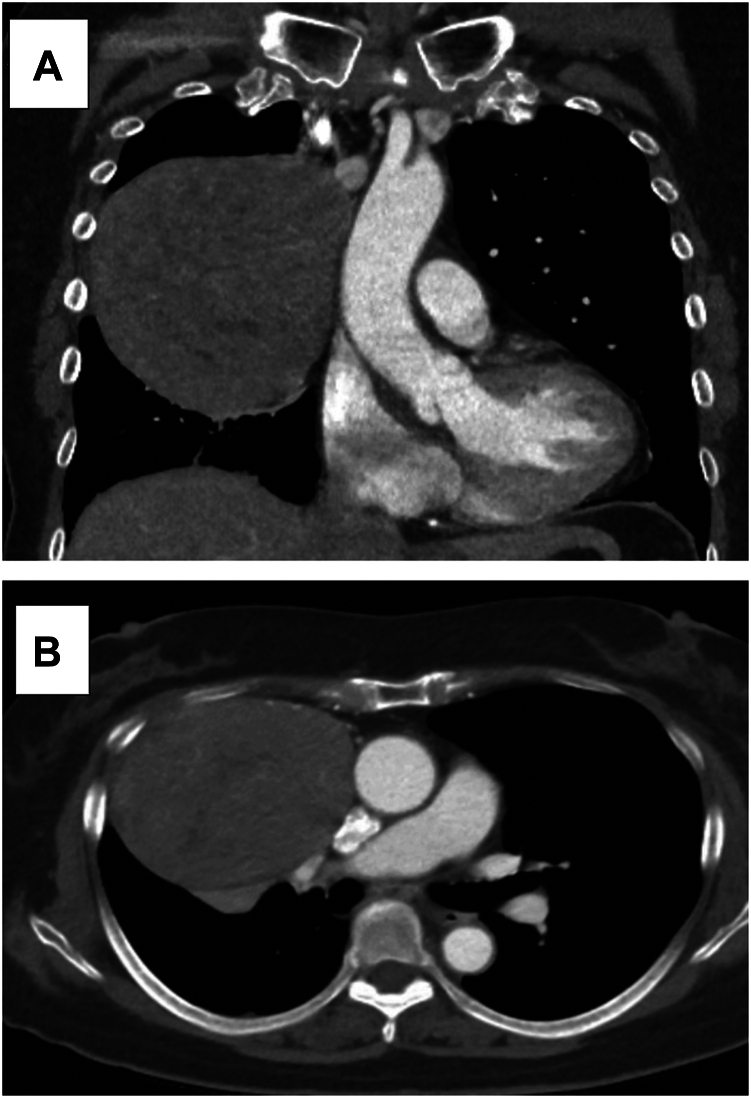
Preoperative computed tomography imaging of the thymoma. (A) Coronal view. (B) Axial view.

The patient underwent a robot-assisted right VATS and en bloc robotic thymectomy in May 2023 by the da Vinci Si Surgical System (Intuitive Surgical). Before incision, large-bore intravenous access was established in the lower extremities because of concern for SVC compression. A camera port was placed in the fifth intercostal space along the anterior axillary line (Figure 2). Entry into the pleural space revealed the mass abutting the chest wall; however, carbon dioxide insufflation facilitated displacement of the tumor, allowing placement of additional working and assist ports.

**Figure 2:**
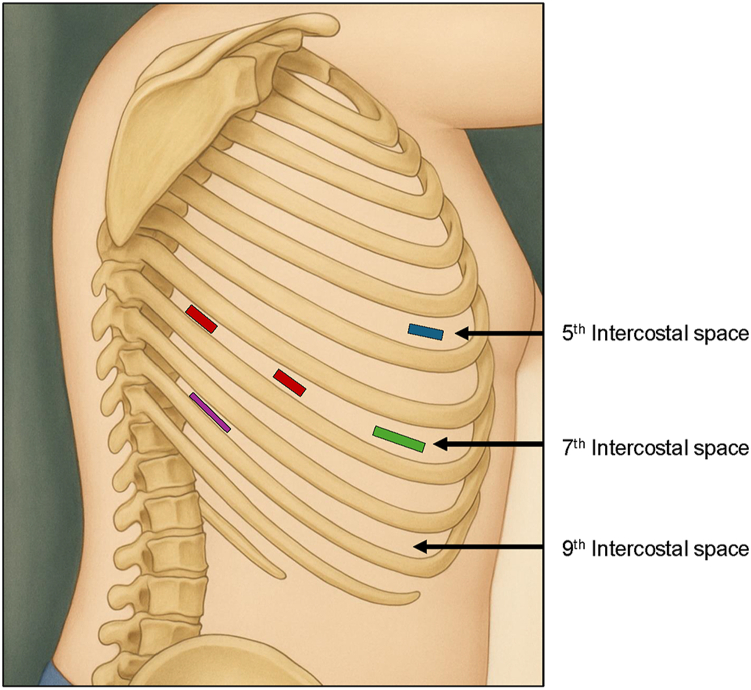
Robotic port placement for right-sided robot-assisted thymectomy of 13-cm tumor. Blue box: 8-mm camera port. Red boxes: two 8-mm working ports. Green box: 12-mm anterior working port. Purple box: 12-mm assist port.

The tumor was compressible, permitting controlled retraction. Mediastinal adhesions were dissected from the mass with the robotic bipolar grasper and vessel sealer, progressively increasing mobility. By rearranging the camera, working, and assist ports into different configurations, we optimized visualization and access to different parts of the mass to facilitate dissection. Alternating between 30° and 45° cameras provided excellent views behind the mass, enabling subsequent mobilization off the SVC. Although circumferentially adherent to the SVC and innominate junction, the tumor was not invading either structure.

Dissection continued inferiorly with anterior traction of the mass. Adhesions involving the right middle lobe were divided with the bipolar grasper, providing complete mobilization from the lung hilum. The tumor was then fully free from mediastinum and lung.

Because of the mass’s size, a mini–anterior thoracotomy (10 cm) was created by extension of an assist port to extract the tumor. A Tuffier retractor increased exposure and aided in extraction. The mass was placed in a large laparoscopic specimen bag, typically used for splenectomy, and removed through the miniature thoracotomy. Visual inspection confirmed phrenic nerve integrity, and a 28F chest tube was placed.

Final pathologic examination confirmed a 13 × 11 × 5-cm, pT1a Nx type AB thymoma with negative resection margins (Figure 3). The patient had an uneventful recovery, with chest tube removal on postoperative day 2 and discharge on day 3. At her 1-month follow-up visit, she reported resolution of dyspnea and return to normal daily life.

**Figure 3:**
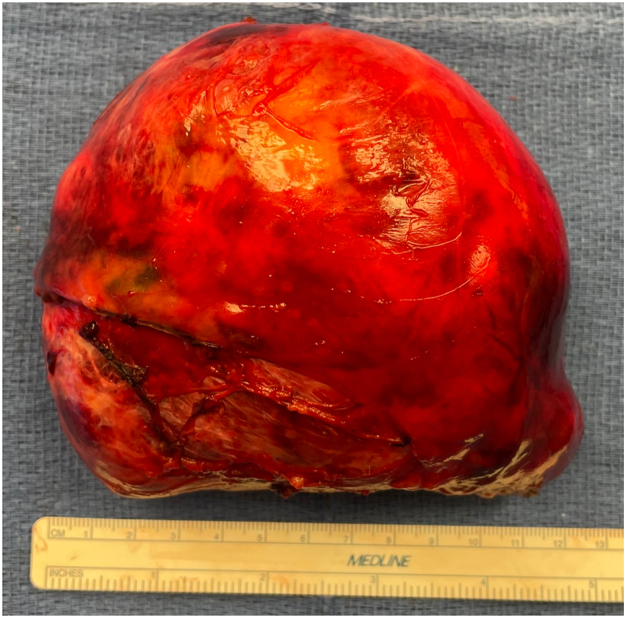
Gross specimen of a 13-cm thymoma resected en bloc through a right-sided robot-assisted thoracoscopic surgery approach.

## Comment

We report the successful completion of an entirely robotic thymectomy for a right-sided 13-cm thymoma in a 68-year-old woman. Although robot-assisted thoracic surgery is now widely used for common procedures, the application of this technique to large anterior mediastinal tumors remains less defined. To date, most large thymomas removed robotically have measured <8 cm,^6^, ^7^, ^8^ and no formal guidelines exist regarding the recommended upper limit for robotic thymectomy.

A primary challenge in these cases is the limited intrathoracic working space, particularly within the confined mediastinum. Even with insufflation and single-lung ventilation, visualization can be impaired by the bulk of a large mass. In our patient’s case, tumor size limited direct visualization. However, the ability to reassign camera, working, and assist ports into various configurations, combined with the enhanced maneuverability of articulating robotic instruments, enabled adequate visualization around the mass and facilitated meticulous dissection of the superior mediastinum.

An important factor in surgical planning and intraoperative decision-making is the tumor’s relationship to adjacent vascular and hilar structures. The robotic platform provided excellent visualization, allowing thorough assessment of the mass and its extension beyond the thymic capsule. We identified that the thymoma was densely adherent to but not invading the SVC and hilar vessels, confirming that an R0 resection was achievable. In this case, palpation to assess for invasion was unnecessary, avoiding the need for an open approach. In some cases, preoperative magnetic resonance imaging may also assist by delineating soft tissue involvement.

In conclusion, thymoma is a rare mediastinal tumor that can grow to considerable size before detection. We present the case of a 13-cm thymoma resected entirely by a robotic approach. Although the tumor’s large size necessitated a miniature thoracotomy incision to permit its extraction, this remained significantly less invasive than a full open approach. We illustrate that tumor size alone should not be a contraindication to robotic thymectomy, particularly when preoperative imaging suggests no invasion of critical structures and intraoperative visualization and dissection can be safely achieved.
